# Machine Learning and Integrative Analysis of Biomedical Big Data

**DOI:** 10.3390/genes10020087

**Published:** 2019-01-28

**Authors:** Bilal Mirza, Wei Wang, Jie Wang, Howard Choi, Neo Christopher Chung, Peipei Ping

**Affiliations:** 1 NIH BD2K Center of Excellence for Biomedical Computing, University of California Los Angeles, Los Angeles, CA 90095, USA; weiwang@cs.ucla.edu (W.W.); jw744@g.ucla.edu (J.W.); cjh9595@g.ucla.edu (H.C.); nchchung@gmail.com (N.C.C.); 2 Department of Physiology, University of California Los Angeles, Los Angeles, CA 90095, USA; 3 Department of Computer Science, University of California Los Angeles, Los Angeles, CA 90095, USA; 4 Scalable Analytics Institute (ScAi), University of California Los Angeles, Los Angeles, CA 90095, USA; 5 Department of Bioinformatics, University of California Los Angeles, Los Angeles, CA 90095, USA; 6 Institute of Informatics, Faculty of Mathematics, Informatics and Mechanics, University of Warsaw, Banacha 2, 02-097 Warsaw, Poland; 7 Department of Medicine (Cardiology), University of California Los Angeles, Los Angeles, CA 90095, USA

**Keywords:** machine learning, multi-omics, data integration, curse of dimensionality, heterogeneous data, missing data, class imbalance, scalability

## Abstract

Recent developments in high-throughput technologies have accelerated the accumulation of massive amounts of omics data from multiple sources: genome, epigenome, transcriptome, proteome, metabolome, etc. Traditionally, data from each source (e.g., genome) is analyzed in isolation using statistical and machine learning (ML) methods. Integrative analysis of multi-omics and clinical data is key to new biomedical discoveries and advancements in precision medicine. However, data integration poses new computational challenges as well as exacerbates the ones associated with single-omics studies. Specialized computational approaches are required to effectively and efficiently perform integrative analysis of biomedical data acquired from diverse modalities. In this review, we discuss state-of-the-art ML-based approaches for tackling five specific computational challenges associated with integrative analysis: curse of dimensionality, data heterogeneity, missing data, class imbalance and scalability issues.

## 1. Introduction

Technological advancements in high-throughput cell biology have enabled researchers to examine the landscape of biomolecules (i.e., DNA, RNA, proteins, metabolites, etc.) associated with a phenotype of interest. Next-generation sequencing technologies [1,2,3] have revolutionized the profiling of DNA and messenger RNA (mRNA), allowing genomes and transcriptomes to be sequenced quickly and economically. Mass spectrometry [4,5] allows us to efficiently identify and quantify proteins, metabolites and lipids in cells, capturing underlying cellular variations in response to physiological and pathological changes. Consequently, large-scale studies on the genome, the transcriptome, the proteome, the metabolome, the lipidome, etc. have created a plethora of data associated with these “-omes” also known as “omics” data. In this regard, machine learning (ML) algorithms [6,7,8,9,10] have been developed to elucidate complex cellular mechanisms, identify molecular signatures, and predict clinical outcomes from large biomedical datasets [11,12]. Traditionally, ML-based single-omics analyses provide assorted perspectives on cellular processes with respect to a particular -ome [13,14,15,16]. However, isolated omics studies frequently fall short when identifying the cause of multifaceted diseases such as cancer [17], cardiac diseases [18], diabetes [19], etc. This evidence suggests that an inclusive view of cellular processes, constructed by integrating information within and across -omes, is required to provide a comprehensive picture of the biological mechanisms [20].

ML-empowered integrative analysis has emerged as a key player in studies involving multiple omics data [21,22,23,24,25]. By analyzing different omics layers together, ML-based integrative methods provide a holistic view of biological processes, offer new mechanistic insights on the phenotype of interest, and facilitate the advancements in precision medicine [26]. For example, Hoadley et al. employed ML-based integrative clustering in a comprehensive study of twelve different types of cancer which resulted in a new molecular taxonomy of diverse tumor types [21]. They integrated genomics, epigenomics, transcriptomics, and proteomics data utilizing cluster-of-cluster-assignments (COCA) to obtain clinically relevant sub-types. In [22], canonical correlation analysis (CCA) with dimensionality reduction was employed for jointly analyzing microRNA (miRNA) and gene expression data. This analysis provided insight into the mechanisms of head and neck squamous cell cancer and its response to treatment via cetuximab. In another study, Arelaguet et al. [23] performed integrative analysis of somatic mutations, RNA expression, and DNA methylation data associated with chronic lymphocytic leukemia (CLL). This study identified new factors predictive of clinical outcome by employing a latent variable modeling approach. To identify markers of body fat mass changes in obesity [24], proteomics and metabolomics data were integrated to create a “transomic” dataset whose individual features went through *z*-score transformation prior to independent component analysis (ICA). It was noted that a combined transomics dataset better discriminates lean and obese subjects as compared to single-omics data. For improving drug sensitivity in breast cancer, genomics, epigenomics, and proteomics, data were integrated using a multiview multiple kernel learning (MKL) approach [25]. This study showed that the predictive performance achieved by multiview learning was found to be better than that obtained by any individual view, where a ‘view’ describes a particular representation of the input data.

Integrative analysis of biomedical data with ML can be performed in a variety of ways. For example, the simplest approach is to construct a large feature matrix by directly concatenating features from different datasets [27]. Each feature may go through *z*-transformation for standardization across all biological samples, followed by ML-based feature selection for molecular signature extraction and biomarker identification. Another common integrative analysis approach is to transform data from heterogenous sources into joint latent profiles. Latent (hidden) profiles are the transformations of data that can capture hidden sources of variation. ML-based clustering is then performed in common latent sub-space for the identification of clinically relevant patient sub-groups [28]. In addition, there are ML-based frameworks that fuse data as a step toward building a model, e.g., multiple kernel learning or network modelling approaches [25,29]. Notably, the accumulation of large biomedical data and the inevitable benefits of studying multiple omics together present new challenges and opportunities for developing novel computational approaches customized for integrative analysis. For example, heterogeneous data with mixed variable types, and missing values in one or more omics can substantially hinder the data integration and analysis. In addition, when integrating multiple omics data, the dimensions of the dataset can grow into hundreds or thousands of variables, while the number of observations or biological samples remains limited. This disparity is called the curse of dimensionality or the *p* >> *n* problem, where *p* is the number of variables and *n* the number of samples. Moreover, the rarity or class imbalance in the data can also lead to results that are biased or less accurate. A class imbalance problem arises when rare events are analyzed and compared against events that happen much more frequently, a common occurrence in omics datasets. Furthermore, standard integrative frameworks may not be suitable for large-scale multi-omics analysis due to computational and storage limitations.

Fortunately, advancements in the field of data science are constantly improving the precision of biomedical research, and machine learning is well poised to enable seamless integration of molecular and clinical data. In addition, deep learning architectures [30,31,32], which better recognize complex features through representation learning with multiple layers, can facilitate the integrative analysis by effectively addressing the challenges discussed above. In this article, we review some of the integrative computational approaches recently proposed for analyzing biomedical data from multiple sources. Specifically, we discuss state-of-the-art ML approaches that can address five important challenges in multi-omics integrative analysis: the curse of dimensionality, data heterogeneity, missing data, class imbalance, and scalability issues.

## 2. Curse of Dimensionality

In the integrative analysis of multi-omics, the number of variables or features to study is increased, but the number of samples is generally the same, since the measurements from multiple platforms essentially belong to the same biological sample. For example, in the stratification of ovarian cancer patients (samples) based on their DNA methylation, miRNA expression and gene expression measurements (variables), the number of variables can be substantially higher than the number of samples (thousands of variables measured on just few hundred patients) [33]. This is the so-called curse of dimensionality or the *p* >> *n* problem in machine learning [25,34]. The increased dimensionality in the number of variables, with the same sample size, makes most ML methods vulnerable to an overfitting problem, i.e., highly accurate on training data but poor generalization on unseen test data [33]. This is due to that fact that the same samples now cover a much smaller fraction of input feature space [7]. The addition of more features may carry new information; however, the benefit of new information can be outweighed by the curse of dimensionality. Dimensionality reduction (DR) is commonly employed in omics studies as datasets from genomics, proteomics, transcriptomics, medical imaging, and clinical trials are frequently faced with the *p* >> *n* problem. DR techniques are employed either as feature extraction (FE) or feature selection (FS) [35,36]. Feature extraction projects the data from high-dimensional space to lower dimensional space, while feature selection reduces the dimensionality by identifying only a relevant subset of original features [34,35,36].

Feature extraction facilitates data visualization, data exploration, latent (hidden) factor profiling, compression, etc. Principal component analysis (PCA), a popular FE method, reduces the dimensionality of the data by orthogonally transforming the high-dimensional features to linearly uncorrelated principal comments (PC). Given orthogonality constraints, the top PCs capture maximal variance in the dataset. PCA in combination with clustering is an intuitive way for exploratory data analysis (EDA), e.g., visualization of sub-groups in a molecular dataset which otherwise are uninterpretable due to high dimensionality. Non-negative matrix factorization (NMF) is another FE method that achieves dimensionality reduction by finding two non-negative matrices whose product approximate the original non-negative matrix. Unlike PCA in which decomposition matrices have both positive and negative values, the resulting matrices from NMF only have positive values; thus, original data is represented only by additive combinations of latent variables. *t*-distributed stochastic neighbor embedding (*t*-SNE) [37] is an FE algorithm increasingly applied for the visualization of high-dimensional data. *t*-SNE is a nonlinear method and hence performs better when the relationships in the data are not linear. The similarity between data points are used to construct joint probability distributions in such a way that the divergence between joint probabilities in low-dimension embedding and original high dimensions is minimal. Autoencoder, a building block of many deep learning networks, can also be employed for nonlinear FE by restricting the number of hidden layer nodes to less than the number of original input nodes [38,39].

Feature extraction approaches are typically used in unsupervised integrative analysis, i.e., when response or group labels are unknown. ML-based FE can facilitate the discovery of disease specific sub-groups in multi-omics studies. In recent years, many feature extraction methods have been proposed for integrative omics exploratory analysis, with many of them based on PCA [40]. For example, multi-omics factor analysis (MOFA) was proposed recently as a generalization of PCA to multi-omics data to identify biomarkers in CLL [23]. Specifically, somatic mutations, DNA methylation and RNA expression were profiled together with ex vivo drug responses and MOFA disentangled sources of systematic variation (latent factors) arising from disease heterogeneity based on the multi-omics data. The latent factors identified by MOFA were shown to be predictive of clinical outcomes. Joint and individual variation explained (JIVE) [41], another extension of PCA, was proposed to identify individual and combined variations between miRNA and gene expression data for the same set of 234 Glioblastoma Multiforme (GBM) tumor samples. JIVE is an integrative EDA method that decomposes a dataset into a sum of three terms: two low-rank approximation terms, one for capturing joint structure across data types and other for capturing structure individual to each data, and a term for residual noise. In order to integrate protein and gene expression datasets from National Cancer Institute (NCI)-60 cell-lines, the multiple co-inertia analysis (MCIA) [42] employed FE methods like PCA on each data set separately to project them to similar (lower) dimensional space for EDA. In MCIA, the diverse sets of variables were transformed to the same scale to easily combine genes and proteins features, providing better biological pathway interpretation. Joint NMF [43] and intNMF [44] performed integrated data exploration with gene expression, DNA methylation and miRNA expression data to facilitate the identification of clinically distinct patient sub-groups by utilizing the NMF concept. In addition, integrative-NMF (iNMF) [45] was able to identify the heterogenous and homogenous factors across different types of data. Non-linear FE techniques including *t*-SNE and autoencoders also play key roles in multi-omics studies. For example, *t*-SNE was employed to facilitate the visualization and clustering in an integrated multi-omics study of transcriptional and epigenetic states in the human adult brain [46], and the integration of single-cell transcriptomic data across different conditions, technologies, and species [47]. In a precision oncology study of cancer cell lines involving gene expression, copy number, mutation status and drug sensitivity data, the dimensionality of the integrated data was effectively reduced by a deep autoencoder [48]. The autoencoder was able to extract cellular state features that were highly predictive of drug sensitivity. Moreover, representation learning [49] or the automatic extraction of meaningful representation of raw data (embeddings), which makes predictive models much more accurate, was also considered for integrated analyses [50,51]. For example, representation learning was employed to generate node embeddings that consequently produced informative edges in biological knowledge graphs [50]. Many life sciences databases make their data available as Linked Data, i.e., data having biological entities and their connections standardized with unique identifiers for better interoperability across resources. In [50], Linked Data, biomedical ontologies and ontology-based annotations were integrated, facilitating functional prediction and the predictions of protein–protein interaction (PPI), drug target relations, candidate genes of diseases, etc. In another study [51], a Multi-view Factorization Autoencoder was proposed for integrating multi-omics data with domain knowledge. This deep representation learning method effectively tackled the *p* >> *n* problem in datasets, and learned feature embedding and patients embedding simultaneously.

In biomedicine, ML-based feature selection methods are frequently applied to identify small subsets of key molecules or molecular signatures [33,52,53,54,55]. FS methods are classified into three main types:

(1) Filter methods,
(2) Wrapper methods,
(3) Embedded methods.

Filter methods are used to select a subset of relevant features independent of any model. Many of the filter methods are univariate and provide statistical test scores for each feature-outcome combination. Examples in this category include ANOVA, Pearson’s correlation, information gain (IG), etc. In addition, maximal-relevance and minimal-redundancy (mRMR), correlation-based FS (CFS) and ReliefF [56,57] are some advanced filter methods which consider feature combinations. For example, mRMR identifies features which are most relevant to the outcome but are not highly correlated among themselves [56]. Wrapper methods try to search for the best feature combination by training a particular predictive model repeatedly for various feature subsets and keep aside the best or worst performing subsets. Therefore, wrapper methods provide the best performing feature combination on that predictive model. Recursive feature elimination (RFE) [58], boruta [59], and jackstraw [60] are popular wrapper methods that repeatedly construct a model (e.g., random forest) and remove features with low weights. Whereas Boruta selects features with critically large variable importance measures in Random Forest, the jackstraw methods identify statistically significant features with respect to latent variables. Wrapper methods can be computationally expensive on a large dataset. Embedded methods are in between filter and wrapper methods in terms of computational complexity. These are the algorithms with built-in feature selection methods, i.e., they perform feature selection as a step toward predictive model building. Least absolute shrinkage and selection operator (LASSO) is a popular embedded FS method due to its simplicity. It is essentially a linear regression method with an *L*1-penalty (regularization) which shrinks many of the coefficients to zero. The features with non-zero coefficients in LASSO are considered relevant variables. However, when the features are correlated, LASSO tends to randomly pick only one feature. Various modifications are proposed to circumvent this problem, including stability selection [61,62,63] and elastic net [64]. Stability selection performs random subsampling and constructs many models on these bootstrap samples. Elastic net strikes the balance between *L*1 and *L*2-regularized regression penalty terms, with *L*1-penality preferring a parsimonious model and *L*2-penality retaining some correlated features such as co-expressed molecules.

Feature selection is generally employed in supervised ML-based integrative analysis (response or group labels are known) including classification and regression applications. In multi-omics studies, FS are commonly employed on each omics dataset prior to integration as datasets are high-dimensional and all the variables in individual datasets may not be informative [65,66,67]. This reduction in the number of variables as a pre-processing step attenuates noise prior to integration [67,68,69,70]. In [71], supervised feature selection for multi-omics data was proposed for Cox regression analysis that identified more true signature genes in cancer prognosis. In [70], an mRMR-based feature selection method was developed to identify epigenetic markers from cancer datasets using gene expression and methylation data. The markers identified through this approach were most relevant and least redundant in prostate carcinoma and leukemia datasets. mRMR was also employed to identify key features in predicting ovarian cancer grade or patient survival using concatenation of genomic, imaging, and proteomic data [72]. In [73], various FS methods including CFS, IG, ReliefF, fast clustering-based feature selection algorithm (FAST) and support vector machine based on RFE (RFE-SVM) were employed to identify features with the highest classification accuracy, in the identification of breast cancer sub-types using protein, gene expression and methylation data. Wrapper and embedded FS methods are multivariate, i.e., they can extract relationships among different features and hence particularly suited to multi-omics studies. RFE is one of the commonly used wrapper FS algorithms in biomedicine [52,53,58,74] and has been recently applied to integrative analysis [33]. In [69], mixOmics R package incorporated *L*1-penalized embedded FS into various supervised omics-integration methods to enable molecular signature extraction. In addition, *L*1-penality based regularization was implemented in unsupervised integrated clustering [28,75], as well as in the integrated predictive modelling framework to allow for genetic feature selection [76].

**Figure 1:**
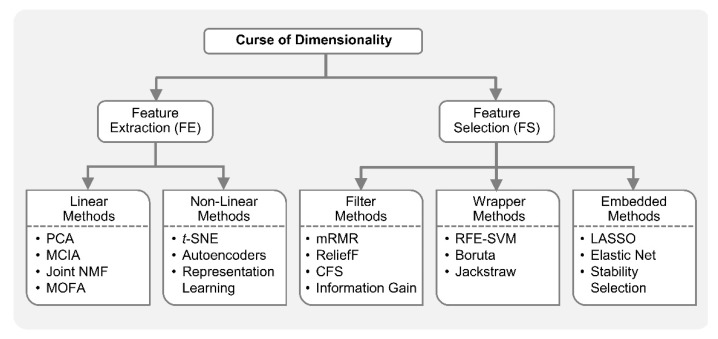
Machine learning (ML) with curse of dimensionality. ML-based dimensionality reduction (DR) approaches, for tackling the curse of dimensionality, can be classified into feature extraction (FE) and feature selection (FS). FE methods project data from a high-dimensional space to a lower dimensional space, while FS methods identify a small relevant subset of original features in order to reduce the dimensionality. Principal component analysis (PCA), multi-omics factor analysis (MOFA), multiple co-inertia analysis (MCIA), and joint non-negative matrix factorization (NMF) are some examples of FE methods applied in integrative analysis. These FE approaches assume linear relationships in the dataset. Nonlinear FE methods also exist including *t*-SNE, autoencoders, representation learning, etc. ML-based FS is broadly divided into filter, wrapper and embedded methods. Filter methods such as maximal-relevance and minimal-redundancy (mRMR), correlation-based FS (FCS), ReliefF and Information Gain are employed as a pre-processing step before training any model, while wrapper methods such as recursive feature elimination-support vector machine (RFE-SVM) and Boruta incorporate a predictive model to judge the importance of features. Embedded methods which include least absolute shrinkage and selection operator (LASSO), Elastic Net, stability selection, etc., perform feature selection as part of the model building process.

Figure 1 shows the taxonomy of ML-based approaches for dimensionality reduction.

## 3. Heterogenous Data

One of the biggest challenges in multi-omics integrated analysis is the heterogeneity of data. Reasons for such heterogeneity include, but are not limited to, substantially different number of variables, mismatched distributions and scaling, diverse data modalities, i.e., continuous signals, discrete counts, intervals, ordered and unordered categorical, pathways, etc. For example, Glioblastoma Multiforme is a highly aggressive type of brain cancer whose prognostic prediction can be improved by considering multiple data types together [77], i.e., clinical data, gene expression, miRNA expression, DNA methylation, and copy number alterations (CNA). However, integration of these diverse data types in a single predictive model is challenging due to heterogeneities mentioned above. In the case of naive data integration, i.e., by concatenating features from different data sources, decision trees (DT) may work well with mixture of continuous and categorical variables. The decision rules in DT are well interpretable, unlike most nonlinear models which are generally considered black-box. In addition, DT has the inherent mechanism of ranking features based on their importance in decision making. However, decision trees are known to suffer from the overfitting problem; consequently, an ensemble of DTs or random forest (RF) [78] is preferred over DT.

Penalized linear models with *L*1/*L*2 regularization also minimize the risk of overfitting and perform feature selection. Therefore, they are also attractive for feature concatenation-based integrative analysis. For example, elastic net [64] was employed for multi-omics analysis in drug–response prediction from the collection cancer cell line encyclopedia (CCLE) [79] encompassing 36 tumor types with diverse variables including gene expression, copy number, mutation values, etc. All of these variables were assembled into a matrix and each feature went through *z*-score transformation across all cell lines. As discussed in the previous section, being a penalized linear regression model, elastic net can perform FS-based dimensionality reduction. However, the final list of key predictors obtained using this model (and tree-based approaches) can be dominated by the variables from a dataset with the largest number of variables. One way to overcome this problem is to perform block-scaling [80], i.e., scaling each variable by the inverse of the number of variables in the corresponding data block. Moreover, it was pointed out in [81] that the results obtained by elastic net with simultaneous analysis of various molecular data types in drug-response studies (containing both continuous and binary variables) are usually dominated by gene expression data (continuous variables). Consequently, the TANDEM method [81] employed a two-stage FS approach where the first stage uses all the binary variables, referred to as upstream data, and the second stage uses continuous gene expression variables or the downstream data. The model selected by TANDEM was more interpretable by preferentially focusing on upstream features while maintaining predictive power comparable to other integrative methods.

Simple feature concatenation-based integration is not feasible in many scenarios because different heterogeneities may be present in datasets and are not known *a priori*. Multiple kernel learning (MKL) [82] has become a popular approach to integrate data by calculating individual kernel matrices for each data type and fusing them into a global model. While kernel matrix encodes similarity between samples, different data sources may have different notions of similarity. Therefore, in MKL, data from each source has a separate kernel matrix. MKL [77] was successfully applied to GBM prognosis from different data types including, gene expression, CNA, DNA methylation, etc., employing the simpleMKL algorithm [83]. Similarly, Speicher et al. [84] integrated DNA methylation, gene and miRNA expression profiles using MKL, and later performed unsupervised clustering to discover cancer sub-types. Bayesian multitask MKL, the top performing algorithm, introduced as a result of a collaborative effort between the NCI and the dialogue on reverse engineering assessment and methods (DREAM) project [25], was applied to integrate data from different profiling sources including, CNA, DNA methylation, gene expression, reverse phase protein array (RPPA), etc., for predicting drug sensitivity in breast cancer cell lines. It employed a Gaussian kernel for real-valued data and the Jaccard similarity coefficient for categorical data. The Multitask MKL algorithm integrated different views from different data types by constructing a global similarity matrix as a weighted sum of the view-specific kernel matrices, where kernel weights reflect the relevance of each view.

Network-based approaches for integrative analysis can also leverage the concept of similarity fusion. Similarity network fusion (SNF) framework aggregated mRNA expression, DNA methylation and miRNA expression data for cancer patients, and used networks as a basis for integration [29]. SNF fused individual similarity networks obtained from different data sources to obtain single similarity network that captures complementary information. It employed scaled exponential similarity kernel in which Euclidean distance was used for continuous variables, chi-squared distance for discrete variables, and agreement-based measure for binary variables. Recently, GloNetDRP [85] was proposed, which built a heterogenous network using cell-line similarity networks from omics data of cell lines, and drug similarity network by exploiting chemical similarity between drugs. Probabilistic graphical models (PGMs) [86] are also a good candidate to integrate mixed data types [87]. For example, in a study of long-term body weight change in the general population [88], a multi-omics partial correlation network was constructed by first employing weighted correlation network analysis (WGCNA) [89] on metabolomics and transcriptomics data separately, and then integrating them using Gaussian graphical model (GGM) [90]. PAthway Recognition Algorithm using Data Integration on Genomic Models (PARADIGM) [91], a factor graph-based PGM approach that was proposed to integrate copy number and gene expression with curated pathway information from NCI, provides patient-specific inference of genetic pathway activities. PARADIGM inferred cellular activities helped classify patients into clinically relevant sub-groups. In [92], sparse graphical models were proposed for accurate group-wise expression quantitative trait loci (eQTL) mapping, by capturing the joint effect of a set of single-nucleotide polymorphisms (SNPs) on a set of genes. This approach used two types of hidden variables, one extracted set associations between SNPs and genes, and the other extracted confounders. Recently, a Network-based Integration of Multi-omics Data (NetICS) [93] method was proposed to prioritize cancer genes by integrating heterogenous multi-omics data into a directed functional interaction network. This interaction network expresses the directionality of the interactions, which is essential as it can explain how aberration events in one gene or miRNA can lead to expression changes of its interaction partners in the network. In addition, heterogenous information networks (HINs) [94,95] which capture multi-level interactions in heterogenous datasets can play important roles in integrative analysis of biomedical data. For example, HeteroMed [96], extracted latent low dimensional embeddings form EHR data (comprising raw text, numeric, categorical formats) for robust medical diagnosis. This method can potentially be extended to the integrative analysis of EHR with other data types.

Another prominent integrative analysis approach involves transforming data from heterogenous sources to latent sub-space, e.g., using PCA or NMF, then performing joint latent analysis or integrative clustering [44,45,97]. This approach allows joint modeling, with a combination of distributions, to include different variable types like continuous (Gaussian), binary (Bernoulli) and count (Poisson) [23]. An integrative clustering method iCluster [28], based on latent variable modelling, was proposed to identify clinically relevant disease sub-types in latent sub-space from two cancer datasets; breast cancer and lung cancer [28] as well from Glioblastoma dataset [75]. Instead of finding clusters of tumor sub-types for each dataset separately and later manually integrating the results, iCluster allowed automated integrated cluster assignment and performed dimensionality reduction simultaneously. This was achieved by leveraging the connection between PCA, latent variable modelling and LASSO-type penalty. Recently, iCluster was upgraded to iCluster+ to incorporate diverse data modalities including, binary, categorical and continuous values such that somatic mutation, CNA and gene expression were integrated and distinct tumor sub-groups were identified [75]. To achieve this iCluster+ assumed different distribution for different data types, e.g., Poisson, normal linear, logistic, multilogit, etc. Recently, the Scluster method had been shown to outperform iCluster and SNF methods in identifying cancer sub-types by jointly analyzing mRNA expression, miRNA expression, and DNA methylation data [97]. A latent factor-based clustering method referred to as mixed variable restricted Boltzmann machine (MV-RBM) [98] was proposed to aggregate data from highly heterogenous sources including demographics, diagnosis, pathologies and treatments in diabetes mellitus studies. With MV-RBM, the datasets were aggregated into latent profiles (homogenous representation), and these profiles facilitated the extraction of patient sub-groups by performing unsupervised affinity propagation (AP) clustering [99]. This approach has the potential to be extended to multi-omics integrative analysis.

**Figure 2:**
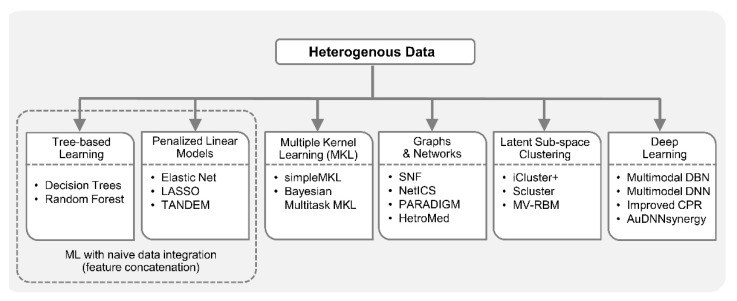
Machine learning with heterogenous data. ML algorithms can handle heterogenous data in different ways. For naive feature concatenation-based data integration, tree-based methods (e.g., decision trees and random forest), and penalized linear models (e.g., elastic net and LASSO) can be employed. A two-stage elastic net-based approach like TANDEM is useful if data sources with continuous features (e.g., gene expression) dominate the data sources with binary features (e.g., mutation). Multiple kernel learning (MKL), a robust integrative analysis approach with heterogenous data, employs different kernels or similarity functions for data from different sources and fuses them into a global matrix. Bayesian multitask MKL and simpleMKL are notable examples in this category. Network fusion methods such as similarity network fusion (SNF) employ similarity network for each data type and fuse heterogenous networks. PAthway Recognition Algorithm using Data Integration on Genomic Models (PARADIGM) can incorporate different heterogenous data including gene expression, copy number and curated pathways. Network-based Integration of Multi-omics Data (NetICS) integrates multi-omics data on a directed functional interaction network. Heterogenous information networks like HetroMed can handle raw text, numeric, and categorical data in electronic health records (EHRs) for medical diagnosis. Integrative methods including iCluster+, Scluster and mixed variable restricted Boltzmann machine (MV-RBM) first transform data from heterogenous sources into latent sub-space, and then perform clustering on the latent profiles. Deep learning models such as improved Clustering and PageRank (CPR), Deep Neural Network Synergy model with Autoencoders (AuDNNsynergy), multimodal deep belief networks (DBN) and deep neural networks (DNN) have been employed to perform integrative analysis of heterogenous data by learning complex features through data transformations at multiple layers.

Deep learning approaches have been getting attention from biomedical researchers to integrate heterogenous data. Specifically, in [100], omics data from multiple sources (gene expression, miRNA expression, and DNA methylation) were combined with clinical data to perform integrated clustering based on multimodal deep belief networks (DBN) [101]. Multimodal DBN is a network of stacked RBMs that seamlessly handles continuous and categorical data, and helps in discovering disease sub-types in cancer patients. In addition to integrative clustering, this method can identify signature genes and miRNAs that may play key roles in the pathogenesis of different cancer subtypes. In [32], a deep learning-based method was proposed to predict cancer prognosis using CNA, DNA methylation, gene expression, and somatic mutation data. This method is an extension of Clustering and PageRank (CPR) algorithm [102] to address the heterogeneity in multi-omics cancer datasets. In [103], three separate deep neural networks (DNN) were trained on gene expression, copy number and clinical data, respectively, for prognosis prediction of human breast cancer. Later, score level fusion was performed to get final multimodal deep network. Hepatocellular carcinoma (HCC) is the most prevalent type of liver cancer in the U.S. and to better understand HCC heterogeneity among patients using gene expression, miRNA expression, DNA methylation and clinical information, a deep learning framework was proposed [104]. This framework employed an autoencoder to perform nonlinear FE on the heterogenous data, which resulted in the aggregation of genes that share similar pathways. Autoencoder transformations led to the discovery of two liver cancer sub-types with significant differences in survival. Recently, a Deep Neural Network Synergy model with Autoencoders (AuDNNsynergy) was proposed that integrated multi-omics with chemical structure data to accurately predict drug combinations in cancer therapy [105]. This model utilized three autoencoders for gene expression, copy number and mutation data. A deep neural network combined the output of three autoencoders with physicochemical properties of drugs, predicting synergy value of given pair-wise drug combination against specific cancer cell lines. Figure 2 lists diverse ML-based approaches available for integrative analysis from heterogenous data.

## 4. Missing Data

Data acquired from high-throughput omics platforms are known to have missing observations due to various reasons, such as low coverage of next-generation sequencing, low sensitivity in protein and peptide detection, and faltered metabolite measurement by tandem mass spectrometry, etc. [106,107]. The problem of missing data is exacerbated in multi-omics studies as there can be more samples with missing values [108]. For example, a CLL study involving simultaneous analysis of DNA methylation, somatic mutation and gene expression measurements against drug response can have up to 40% of the biological samples with some but not all omics data, i.e., missing values in 40% of the samples [23]. Given that the biological samples are the same, it is statistically plausible to infer missing values in one omics from observed values and in other omics by exploiting any existing correlations found through complete cases. Complete case refers to the samples with measurements available on all variables under consideration [106,107,109,110]. Generally, most modern missing data methods focus on *item non-response* case, i.e., when data is missing on some variables for some biological samples [106,111,112]. Other cases include data missing on all variables for some biological samples, known as *unit non-response*, and data missing on a variable for all samples, known as *latent variable*. Missing data methods should be able to maximally utilize the available information, properly estimate the uncertainty in missing values and minimize bias [113].

Most statistical approaches rely on certain assumptions to tackle the missing data problem [111]. Suppose data is missing on variable *Y* while another variable *X* is always observed. The strongest assumption is that data is missing completely at random (MCAR), meaning that the probability of missingness on *Y* does not depend on *X* as well as on *Y* itself. For example, in a clinical study, it may be difficult to obtain a particular test result because the test itself is costly, hence it is only available for 30% of the samples. For the remaining 70%, the data is MCAR. Note that, if data is MCAR, the complete data subsample is just a random sample from the original target sample. The MCAR assumption is required by conventional methods, which is frequently violated in practical applications. However, most modern approaches work well with a weaker assumption of data missing at random (MAR). MAR assumes the probability of missingness on *Y* does not depend on *Y*, after controlling for the observed variable *X*, i.e., once dependence on *X* is adjusted, the probability of missingness on *Y* does not depend on *Y* itself. Again, consider the clinical study example in which cholesterol levels are missing for many subjects and the probability of missingness depends on subject’s sex, i.e., females may be less likely to report cholesterol levels than males. However, within each gender type, subjects with higher cholesterol levels are neither more nor less likely to report than subjects with lower cholesterol levels. We can say that the cholesterol level variable has data missing at random because, after adjusting for subjects’ gender, the missingness of the cholesterol level variable does not depend on whether the cholesterol level is high or not. MCAR is a special case of MAR, i.e., if data is missing completely at random then they are also missing at random. If the data is not missing at random (NMAR) then the missing data mechanism has to be modelled [113,114], i.e., simultaneous estimation of the scientific model and missing data mechanism is required.

The simplest approach to deal with missing data is a complete case analysis also known as listwise deletion. Listwise deletion means that the entire sample is excluded from analysis if data is missing on any variable for that sample. However, it may result in substantial information loss if the missing data percentage is high. In addition to complete case analysis, traditional single imputation methods are also very popular due to their ease of implementation. Any approach which estimates or guesses the missing values is called imputation. Missing values on a variable can be imputed by replacing it with a mean or median of the variable over all the available samples. Imputation based on regression or conditional mean imputation trains any type of regression model for the variable with missing data based on observed values. Subsequently, the model is used to generate predicted values for the cases with missing data. The *k*-nearest neighbors approach is also commonly employed for imputation of missing values.

In multi-omics studies, imputation based on *k*-nearest neighbors for profiles and genes expression [76], autocorrelation with cubic interpolation for spectral analysis of time series molecular data [115], fully conditional specification (FCS) for metabolite concentrations [88], etc., were employed for one or more data types separately, prior to integration [33]. In [107], stochastic gradient boosted trees (GBT) was employed to predict protein abundance for undetected proteins by exploiting the nonlinear correlations between available transcriptomics and proteomics data [107,116]. A multi-omics imputation method that considers correlations across microRNA, mRNA and DNA methylation data, and iteratively performs self-imputations (with features from same omics data) and cross-imputations (with features from different omics data) was implemented by employing an ensemble regression framework [110]. In general, it is recommended that any deterministic imputation should be done multiple times to account for the uncertainty in imputed values [113,117]. Consequently, various multiple imputation (MI) methods have been proposed [118,119,120,121]. In MI, instead of imputing single value for each missing data point, multiple values are imputed, resulting in multiple completed datasets rather than just one [122]. The observed values are the same in each dataset, but imputed values are slightly different. This difference is generally achieved by making random draws from error distribution of the regression model and adding those random draws to the values predicted by that regression model. Moreover, instead of explicitly assuming that regression parameters are true parameters and not estimates, these parameters can be randomly drawn from their posterior distribution for each dataset separately [113,118,119,120]. MI is an attractive approach for missing data because of its sound statistical properties and robustness established by extensive simulations.

Recently, a MI-based approach, referred to as MI for multiple factor analysis (MI-MFA), was proposed for multi-omics data integration [123]. MI-MFA used hot-deck imputation, which is a non-parametric method commonly used in big surveys due to its scalability to a large number of variables with missing values. To perform hot-deck imputation, the missing value on a variable is replaced with an observed value from a similar sample or donor. Some other popular iterative MI methods include Markov-chain Monte Carlo (MCMC) [118], fully conditional specification, also known as, sequential generalized regression or multivariate imputation by chained equation (MICE) [119] and AMELIA II [120]. MCMC is a general method used in Bayesian statistics for various applications. MCMC assumes a comprehensive joint distribution of all variables with missing data, generally applied under multivariate normal assumption. A key feature of MCMC is that imputed values are never used as the basis for predicting other missing values, i.e., imputations are only performed based on observed data. Given all assumptions are met and enough iterations are run, MCMC is guaranteed to converge to the correct posterior distribution for the imputed values. However, due to multivariate assumption and having one comprehensive model for all of the variables, MCMC may not be preferred for datasets with both quantitative and categorical variables. MICE, also an iterative algorithm like MCMC, is preferred in mixed-type datasets which builds a separate regression model for each variable depending on its type. MICE can also incorporate methods for imputing data that are not normally distributed [121]. Unlike MCMC, MICE does not have any theoretical proof of convergence and it can also be computationally much more expensive than MCMC. There is a risk of overfitting associated with any data imputation technique, but MI methods are generally less prone to this problem than single imputation methods [124]. However, most software packages available for MI methods assume data is MAR. When data is NMAR, extra care must be taken in date imputation to avoid overfitting and the introduction of bias in downstream analyses. Various plausible models should be tried, e.g., MI with pattern–mixture models [125]. This should be accompanied by sensitivity analysis to verify the consistency of the results across models.

In addition to MI methods for missing data, there are several ways to get maximum likelihood estimates with missing data based on multivariate assumption, including expectation–minimization (EM) and direct maximization of the likelihood or full information maximum likelihood [114,123]. Maximum likelihood is a general method commonly employed for parameter estimations in linear models. Compared to MI, maximum likelihood approaches generally have more rigorous mathematical proofs related to parameter estimation with missing data. Maximum likelihood chooses as parameter estimates those values which maximize the likelihood function, given the observation, i.e., maximize the probability of observing the data. The main disadvantage of maximum likelihood is that it is restricted to the type of model you want to estimate, e.g., linear or logistic regression. To obtain maximum likelihood with missing data, you need software that is specifically designed for the model you want to estimate, which is not always available, whereas MI methods are more general and can be employed in different types of analyses. There are many software packages that automatically generate multiple imputed datasets and combine results from multiple linear regression analyses in programming software R including, jomo, mice, Amelia II, etc. [120,126]. Notably, most missing data approaches exist only for linear analysis.

For nonlinear analysis with missing data, a two-stage MI and learning workflow based on Gaussian mixture model (GMM) and extreme learning machine (ELM) is available [127]. In order to include nonlinearity in MICE imputation, a random forest-based MICE algorithm was proposed for epidemiological study of angina patients [128]. This method can accommodate nonlinearities in the datasets and provide better parameter estimates, confidence intervals under MAR assumption. Deep learning techniques were recently applied to handle missing data in biomedical datasets [129,130,131,132]. The success of many of these data imputation methods can be contributed to autoencoder-based nonlinear FE. In [129], a multilayer autoencoder with dropout-based imputation on EHR datasets for amyotrophic lateral sclerosis (ALS) clinical trials was shown to outperform popular MI techniques including MICE. In addition, a denoising autoencoder (DAE)-based MI (MIDA) was also proposed very recently [130]. MIDA outperformed MICE algorithm on multiple datasets from various domains including bioinformatics.

AutoImpute [132], inspired by recommender systems (collaborative filtering) in information retrieval, is an autoencoder-based method for single cell RNA-seq (scRNA-seq) gene expression imputation. This method learns the distribution of scRNA-seq data and imputes the dropout (i.e., missing) gene expressions accordingly. In scRNA-seq analysis with missing data, matrix factorization-based imputation techniques are also popular in replacing the dropout with non-zeros values. For example, adaptively-thresholded low-rank approximation (ALRA) [133] computed a low-rank approximation of original matrix with missing data using singular value decomposition (SVD), followed by a thresholding to ensure that the biological zeros are preserved and technical zeros were imputed. SVD-based imputation techniques have traditionally been used in biomedical datasets due to their simplicity and superior performance rather than simple mean imputation [134]. Recently, the Sparse Recovery (SparRec) framework [135], also inspired by a low-rank matrix factorization model, was proposed for genetic data imputation for genome-wide association study (GWAS). It is a flexible imputation method that can be applied to large-scale meta-analysis, even without a reference panel. Sequencing To Imputation Through Constructing Haplotypes (STITCH) [136] is another notable imputation technique for quick and cost-effective genotyping from sequence data without reference panel. The imputation in STITCH is based on hidden Markov model (HMM) and EM algorithms.

**Figure 3:**
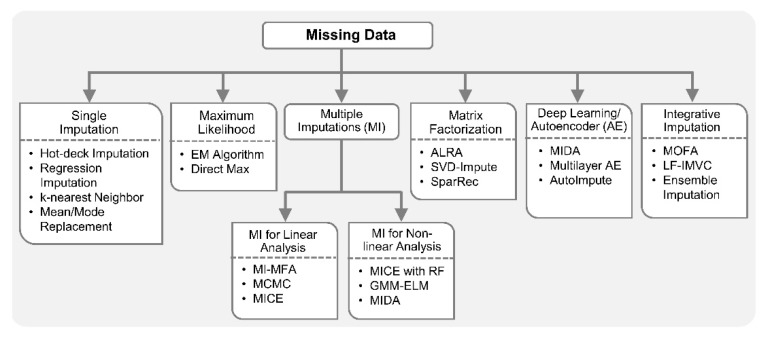
Machine learning with missing data. Conventional single imputation methods for handling missing data include replacement with mean or mode values, hot-deck imputation, regression imputation, *k*-nearest neighbor, etc. Maximum Likelihood approaches including those based on an expectation-minimization (EM) algorithm and Direct Maximization have attractive statistical properties compared to the conventional methods that often result in biased parameter estimates. Multiple imputation (MI) methods like Markov-chain Monte Carlo (MCMC) and multivariate imputation by chained equation (MICE) are also statistically robust, compared to conventional single imputation methods, as they take into account the uncertainty in the imputed values. MI for multiple factor analysis (MI-MFA) tackles the missing data problem in multi-omics analysis by performing MI based on hot-deck imputation. MI for nonlinear analysis can be performed using random forest (RF) and extreme learning machine (ELM). Adaptively-thresholded low-rank approximation (ALRA), singular value decomposition (SVD)-impute and SparRec methods employ matrix factorization for data imputation. In addition, imputation methods based on autoencoder and deep learning like denoising autoencoder-based MI (MIDA), AutoImpute and multilayer autoencoder (AE) have been proposed for high-dimensional datasets with missing data. Recently, integrative imputation methods such as ensemble regression imputation, multi-omics factor analysis (MOFA) and Late Fusion Incomplete Multi-View Clustering (LF-IMVC) are also available.

In multi-omics and clinical big data analytics for precision medicine, missing data is a challenging problem [106,117] and conventional methods are prone to adding biases. Specialized integrative methods, such as ensemble regression imputation [110], can perform integrative imputation by combing the estimates from individual omics data itself as well as other omics. Similarly, MOFA [23] can leverage information from multiple omics layers to accurately impute missing data in integrative analysis. Specifically, it discovers latent factors by means of multi-omics FE and uses those factors to impute missing data. In addition, recently proposed Late Fusion Incomplete Multi-View Clustering (LF-IMVC) [137] is also attractive for multi-omics studies with missing data, where each data source with missing values can be treated as an incomplete view. LF-IMVC employs a kernel matrix for each view, and performs imputation and clustering simultaneously. To this end, modern statistical and machine learning methods such as MI, maximum likelihood, matrix factorization, autoencoders and integrative imputation methods can play key roles in facilitating integration of datasets with different missingness patterns. Figure 3 summarizes statistical and machine learning based solutions for handling missing data.

## 5. Rarity and Class Imbalance

In omics studies, ML-based models are often faced with the rarity in the target class or the class imbalance problem [12,138]. For example, a machine learning classifier trained to predict the location of enhancer in the genome suffers from the class imbalance problem, i.e., the dataset has many more negative samples (non-enhancer) compared to positive samples (enhancer) [12,139]. Similarly, ML-based contact map prediction in a protein structure dataset also suffers from the imbalance problem because of the sparseness of the contacts, i.e., of all possible amino acid pairs in a protein, only about 2% are in contact [140]. Prediction of post-translation modifications (PTM) sites in a protein sequence also encounters the same problem as occurrence of PTM is a sparse event [141], i.e., most of the amino acid residues are not modified. Other examples of the imbalanced problem in omics studies include prediction of protein-DNA binding residues from primary sequences [142], miRNAs identification [143], mutations incidence prediction [144], DNA methylation status/sites prediction [145,146], PPI sites prediction [147,148], identification of antimicrobial peptides (AMP) functional types [149], etc. In addition, the class imbalance problem in clinical datasets is prevalent due to the intrinsic imbalance in case-control pairing. Experimentally, it is often challenging and costly to generate data from a treatment group as compared to a control group [150,151]. Biomedical datasets belonging to the study of rare diseases or events are often severely imbalanced and most ML algorithms are not appropriate in such cases [152,153,154].

Despite the pervasiveness of imbalance in class distribution in real-world datasets, most ML classifiers including SVM, RF, and artificial neural networks (ANN) assume balance class distribution. This assumption means that the number of samples from each group or class is approximately the same (all categories are equally represented) [152,153]. Therefore, these classifiers overestimate the majority class and potentially ignore the minority class completely. Ironically, in most cases, minority class is the target class, e.g., a rare disease sub-type. A classifier trained on a rare disease dataset with 10,000 samples from the control group and 100 samples from the disease group can achieve 99% accuracy by predicting everything belonging to the majority class, without even detecting rare disease [155]. To tackle this problem, ML methods which are aware of the skewness in data or class imbalance learning (CIL) methods have been proposed. Broadly, CIL methods are divided into three categories; data sampling, algorithm modification and ensemble learning. Data sampling methods are frequently employed in biomedical domains because of its simplicity [145,147,149,156,157,158]. Data sampling approaches tackle class imbalance by balancing the dataset prior to applying the ML classifier. The majority class can be undersampled by removing some of the samples randomly, i.e., random undersampling (RUS) or informatively using one-sided selection [159]. New minority class samples can be synthetically created using the synthetic minority oversampling technique (SMOTE) [154]. Recently, a combination of undersampling and oversampling is becoming popular to tackle the imbalance problem more effectively, by overcoming the limitations associated with individual data sampling approach [145,151].

Algorithm modification approaches modify the machine learning algorithm, while still using the original imbalanced dataset. For example, cost-sensitive learning methods apply higher misclassification weight (cost) to minority class samples compared to majority class samples. Cost-sensitive weighting are frequently incorporated in SVM, ANN and boosting learning theory to tackle class imbalance [160,161,162]. Cost-sensitive learning approaches such as SVM_Weight [160] and WeightedELM (WELM) [163] are generally much more efficient than data sampling approaches, and hence attractive for big datasets [152]. However, they require theoretical understanding of the algorithm, as opposed to randomly undersampling the majority class [164]. Lastly, ensemble learning methods generally achieve better generalization performance than data sampling and cost-sensitive CIL methods [148,163,165,166]. In various clinical scenarios, it is a common practice to seek opinions of multiple doctors who are experts in the field. The final decision, for a particular treatment, is thus made by consulting a committee of experts and combining their opinions. In the context of ML, ensemble learning systems play a similar role [167,168]. The majority class is divided into several subsets (with or without replacement), each individual classifier in the ensemble is trained on all the minority class sample and a subset of majority class, and a final decision is based on aggregating the predictions from individual classifiers [139,142,148,163,167]. EasyEnsemble, Balanced Cascade, and ensemble WELM are some examples of ensemble methods for CIL [163,167]. It is important to mention that ensemble learning is a broad category of ML approaches that is not limited to class imbalance learning applications. For example, it has also been employed in integrated frameworks proposed for heterogenous and missing data [25,110].

Although many CIL methods exist for single omics studies, researchers have recently started developing imbalance-aware integrated omics analytical frameworks [69,169,170,171,172]. In [170], extensive simulations based on different integration algorithms and evaluation measures reveal that composite association network, relevance vector machine (RVM) and Ada-boost RVM were less influenced by class imbalance compared to other graph-based or kernel-based integration algorithms. A cross-organism PPI predive modelling was proposed based on tree-augmented naive Bayes (TAN) classifier (TAN relaxes the string independence assumption of NB) that integrated microarray expression and gene ontology (GO) values [173]. PPI data is highly imbalanced since the number of interacting proteins is much smaller than non-interacting protein pairs. Specifically, the imbalance ratio (IR) of non-interacting to interacting protein pairs was around 20. Dividing the imbalance dataset into 20 balanced datasets with the same positive samples produced better results as compared to imbalanced datasets. In [73], equal-class data sampling was performed to reduce the effects of class imbalance in identifying breast cancer sub-types through the integration of protein, methylation and gene expression data.

A PPI prediction method based on RF was proposed which not only considered affinity purification and mass spectrometry (APMS) data, but also various other indirect features including mRNA co-expression, gene ontologies and homologous protein [174]. This method, referred to as Spotlite, avoided the extreme imbalance in data, first by uniformly sampling the unknown interactions so that the IR is 10. Then, during the training of RF classifier, weights of 10 and 1 were assigned to known and unknown interaction classes, respectively. For automatic function prediction (APF), a cost-sensitive network integration approach unbalance-aware network integration and prediction of protein functions (UNIPred) [175] was proposed to integrate biological networks from different data sources. UNIPred addressed the imbalance between annotated and un-annotated proteins by building a consensus network from multiple protein networks derived from different omics data. MNet [176] builds a composite network by integrating multiple functional networks constructed from different proteomic sources to get a comprehensive view of proteins and predict their functions. The protein function prediction is an imbalanced classification problem and MNet addressed this problem by employing weighted functional labels (label represents distinct protein function), putting more emphasis on the labels that have fewer member proteins. A cost-sensitive SVM approach was proposed for diagnosing pancreatic cancer by integrating miRNA and mRNA expression data [177]. The dataset was imbalanced as there were 104 pancreatic ductal adenocarcinoma (PDAC) tissues and 17 benign pancreatic tissues. Therefore, class specific weights in SVM for cancer and normal samples were set to 1 and 6.117647 (104/17), respectively. Using their integrated approach, they were able to identify 705 multi-markers for 27 miRNAs and 289 genes as promising potential biomarkers for pancreatic cancer. The generalized simultaneous component analysis (GSCA) model, with GDP penalty, was proposed recently for the integrative analysis of gene expression and CNA [178]. This method was found to be more robust against class imbalance problem in CNA compared to iCluster+ method. In [179], authors showed that a simple ensemble learning method can work as well as state-of-the-art data integration methods such as kernel fusion. The ensemble comprised learners which were trained on different views of data and the predictions were combined using weighted majority voting (WMV). The weight was determined using F-score that considered the imbalance between gene classes.

Apart from data sampling, algorithm modification and ensemble learning based methods, some integration frameworks which perform model tuning based on CIL-specific evaluation measures were proposed recently. Traditional evaluation measures like overall accuracy are not appropriate for CIL [172]. The accuracy of the majority class (specificity) and the accuracy of the minority class (sensitivity) should be measured in a balanced way. Therefore, geometric mean (Gmean) of sensitivity and specificity is a commonly used evaluation measure for CIL [153]. Similarly, area under precision-recall curve (auPRC) provides more unbiased evaluation compared to the area under receiver operating characteristic (auROC). Matthews correlation coefficient (MCC) and F-scores also take into account imbalance in class sizes. F-score, which incorporates precision and recall, is a popular evaluation metric in information retrieval community [170]. MCC [14,145] considers true positives, true negatives, false positives and false negatives in its formula. It can have a value between −1 and 1; 1 means perfect prediction, 0 means random prediction and −1 means total disagreement. Balanced error rate (BER) calculates the average proportion of incorrectly classified samples in each class, weighted by the number of samples in each class. To address the imbalance problem in multi-omics predictive modelling, BER was incorporated as an evaluation measure for parameter tuning, through cross-validation, in data integration analysis for biomarker discovery using latent components (Diablo) [69,171]. Diablo is a multi-omics integrative framework which can identify biomarker panels that discriminate between different disease phenotypes. It transforms each omics dataset into latent components, and maximizes the correlations between these components and phenotype of interest. A novel neural network architecture incorporating cross-correlation between different modalities (e.g., gene expression and DNA methylation) was proposed in [172] to classify breast cancer patients. This method, referred to as super-layered neural network architecture (SNN), utilized MCC and F-scores to account for the imbalance in class sizes. In general, most methods and evaluation measures for CIL are proposed for binary class problems, i.e., there are only two categories in the dataset. However, multi-omics data analysis and hypothesis generation may involve more than two classes [66], with a varying degree of imbalance among them [172]. For example, instead of normal vs. disease samples, there can be different types or levels of diseases [72,157,163,172,180]. In recent years, researchers have started focusing on multi-class imbalance problems [152,163,181]. Fuzzy pattern random forest (FPRF) [181] employed multi-class version of F-score and Gmean for robust feature selection in the integrative analysis of an imbalanced Leukemia dataset.

**Figure 4:**
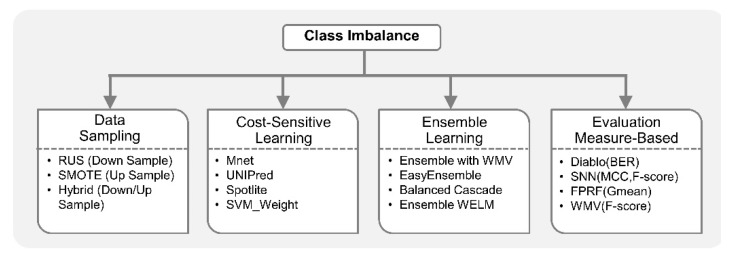
Machine learning with class imbalance. Class imbalance learning (CIL) methods are broadly classified into three types: data sampling, cost-sensitive learning and ensemble methods. Data sampling approaches balance the class distribution by either undersampling the majority class (e.g., random under sampling (RUS)), oversampling the minority class (e.g., synthetic minority oversampling technique (SMOTE)), or a combination of both (hybrid). Algorithm modification methods modify the learning algorithm generally by cost-sensitive weighting (e.g., Mnet, unbalance-aware network integration and prediction of protein functions (UNIPred), Spotlite and support vector machine (SVM)_weight). Cost-sensitive learning assigns a higher misclassification cost to minority class samples compared to majority class samples. Ensemble learning approaches like ensemble with weighted majority voting, EasyEnsemble, Balanced Cascade, and ensemble weighted extreme learning machine (WELM) train multiple classifiers, and aggregate their results to get the final output. Many existing integrative methods tackle imbalance by tuning models based on imbalance-aware evaluation measures. For example, data integration analysis for biomarker discovery using latent components (Diablo), super-layered neural network architecture (SNN), fuzzy pattern random forest (FPRF), and weighted majority voting (WMV) employ one or more CIL-specific evaluation measures like F-score, balanced error rate (BER), geometric mean (Gmean), Matthews correlation coefficient (MCC), area under precision-recall curve (auPRC), etc., instead of classification accuracy, to account for the bias introduced by imbalance in the dataset.

Due to the inherent sparsity in various omics phenomena, rare events in diseases of interest and case-control imbalance in clinical studies, it is anticipated that integrated omics studies will present new challenges in predictive modelling and provide opportunities for researchers to propose specialized CIL algorithms. For example, beyond simple data sampling approaches, biomedical researchers can explore ensemble and algorithmic modification methods that generally have better theoretical foundations, natural scalability to multi-class classification, and lower risks of overfitting and information loss than data sampling approaches. Figure 4 shows categorization of class imbalance machine learning methods.

## 6. Big Data Scalability

**Figure 5:**
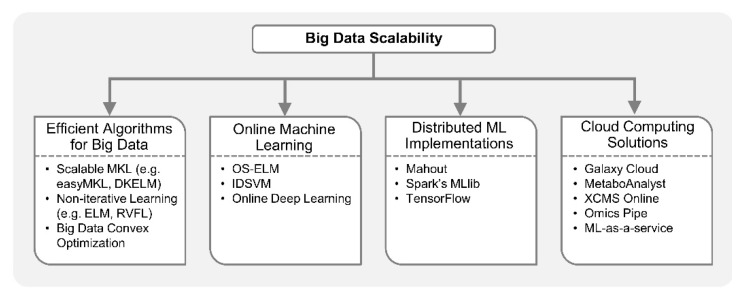
Large-scale machine learning. ML-based integrative analysis can be performed at large-scale by utilizing computationally efficient algorithms proposed for big data, online training algorithms, distributed data processing and computing frameworks, or cloud computing-based solutions. Efficient computational approaches tailored for big data include non-iterative neural networks (e.g., extreme learning machine (ELM) and random vector functional link (RVFL)), scalable multiple kernel learning (MKL) methods (e.g., easyMKL and dual-layer kernel ELM (DKELM)), convex optimization for big data, etc. Online machine learning algorithms including online sequential extreme learning machine (OS-ELM), incremental decremental support vector machine (IDSVM), and online deep learning are attractive for big data applications as they incrementally update the model with small chunks of data, instead of loading entire data in memory and learning all at once. In addition, ML algorithms can now be massively parallelized over a cluster of CPUs or graphics processing units (GPUs) using Spark’s MLlib, Apache Mahout, and Google’s TensorFlow programming frameworks. Cloud computing-based bioinformatics platforms including Galaxy Cloud, MetaboAnalyst, XCMS online, and Omics pipe are useful resources for multi-omics exploratory data analysis (EDA) and ML. Moreover, machine learning-as-a-service is being offered by leading commercial cloud service providers like Amazon, Google, Microsoft and IBM, which can be utilized for implementing ML-based analytical pipelines in large-scale multi-omics studies.

Machine learning algorithms build data driven models whose performance generally gets better with the availability of more data. However, machine learning from big data acquired via multiple high-throughput omics platforms may raise scalability challenges. Implementation of multi-omics analytical workflows based on ML methods is increasingly becoming infeasible on a single computer. However, with the advancement in optimization algorithms for big data, online ML, parallelization of ML algorithms, and cloud computing, large-scale analysis can be performed efficiently on high-dimensional omics datasets. For example, a feed-forward neural network with multiple hidden layers can now be trained to accurately differentiate non-coding RNA types, i.e., circular RNAs (cirRNAs) from long non-coding RNAs (lncRNAs) in just a few hours on a single computer while the MKL method would take four days [182]. This is possible due to the development of computationally efficient training algorithms for neural networks [183,184,185]. Biomedical researchers can achieve large-scale machine learning by leveraging the computational approaches discussed below, as shown in Figure 5.

Various ML methods including ANN, SVM and DT estimate model parameters through iterative procedures; thus, they may not be easily scalable to big data applications. In recent years, there have been many efforts to optimize algorithms for training ML models efficiently on large datasets [183,184,186,187]. For example, non-iterative training algorithms are becoming popular for big data applications [187]. ANN can be trained in a single step without iterative tuning of hidden node parameters, as opposed to a back-propagation (BP) algorithm which is time-consuming, converges slowly, and can be stuck at local minima [183]. Non-iterative solutions for ANN include extreme learning machine (ELM) [188], random vector functional link (RVFL) [189,190], liquid state machine [191], echo state network [192], etc. In most of these methods, weights connecting input layer to hidden layer are randomly assigned, and output weights connecting hidden layer to output layer are determined analytically. Therefore, computational complexity of non-iterative methods is much lower than traditional BP methods for ANN. Furthermore, a highly parallel implementation of ELM for big data has been proposed by employing large-scale optimization [186]. Specifically, convex optimization, a key competent in training many ML and statistical models, is being reinvented for scalability and parallelism in the wake of big data [193]. Recently, methods based on ELM theory have been employed in single omics studies [163,182,194,195,196,197] and may be extended to multi-omics for efficient integrative analyses. Moreover, scalable MKL methods like dual-layer kernel extreme learning machine (DKELM) [198] and easyMKL [199] can be employed in multi-omics integrative analysis since MKL, a popular approach for integrating multiple omics datasets, can be computationally very expensive for large datasets.

Online algorithms are also useful in big data applications, especially when it is computationally infeasible to train models on the entire dataset all at once [200]. They are extremely popular in data stream analytics where the training samples arrive over time, e.g., in online prediction of glucose concentration in Type I diabetes [201]. Instead of retraining the model with the entire dataset every time new samples are received, online learning methods incrementally update the earlier learnt model only with the new samples. Previously learnt samples need not be stored in memory. On the other hand, batch ML algorithms would perform intensive training iterations over the entire dataset every time new samples arrive. In addition, batch learning requires complete datasets to be available in the memory prior to training, which may not be feasible in large-scale applications. Recursive least squares, a sequential (online) implementation of least squares method, is the building block of many online learning algorithms. For example, online sequential extreme learning machine (OS-ELM) [202] is a family of algorithms based on recursive least squares formulation for online training of single hidden layer feedforward networks (SLFNs). OS-ELM based algorithms can learn data one sample at a time or as chunks of samples, and have been employed for nonlinear classification and regression applications. Stochastic gradient decent (SGD), a variant of BP algorithm, is also a popular online optimization algorithm for training ML models [203]. SVM-based online learning algorithms such as incremental decremental SVM (IDSVM) and cost-sensitive learning-based online SVM [204,205] were proposed to address scalability issues in big data applications. Recently, multi-layer or deep online learning methods were proposed for better representation learning with high-dimensional datasets. These deep learning approaches are memory efficient as entire datasets need not be stored in memory, making them attractive for large-scale multi-omics analysis [206,207]. Online learning algorithms are now available for common ML tasks such as classification, regression, feature extraction, clustering, deep learning, etc.

Institutions can also leverage distributed implementations of ML algorithms, on a cluster of computers, when standalone commodity PCs lack the computational power required to learn from big data. For example, the MapReduce [208,209] programming framework provides a distributed platform to process big data in a fault tolerant way and can facilitate the scalability of ML algorithms on large biomedical datasets. Simply put, distributed frameworks like MapReduce and its open-source implementation Hadoop [210] divide the training data into many subsets such that each subset is processed by a single machine or slave. Slave machines perform operations in parallel and results are combined by a centralized master server. MapReduce is a good candidate for scaling those learning algorithms which can be expressed as computing sums of function of training data. Recently, a clustering algorithm KAymeans for MIxed LArge data (KAMILA) [211] was implemented on very large dataset using Hadoop [212]. KAMILA can be useful in multi-omics analysis since it was proposed for mixed-type data (combination of continuous and categorical data) clustering. From the original MapReduce framework, various computational platforms have arisen which are suitable for large-scale ML, such as Apache Spark [213]. These cluster computing platforms efficiently perform multiple iterations of matrix inversions and multiplications which are associated with many ML algorithms. Spark’s MLlib [214] is a suite of scalable algorithms, providing distributed implementations of popular ML methods including regression models, PCA, *k*-means clustering, DT, Naïve Bayes, SVM, etc. Another open-source project that allows distributed implementation of ML algorithms for big data is Apache Mahout [215]. Mahout was successfully employed for scalable feature selection, data sampling and classification in protein structure prediction problems [140]. In addition, Google’s TensorFlow programming model [216] allows parallelism of deep leaning approaches [31] such as convolutional neural networks (CNN) and long short-term memory (LSTM) algorithms, by distributed implementation on many CPUs or graphics processing units (GPUs) for large-scale analysis.

If memory and computational resources required for integrative analysis is beyond what is available in the cluster of a research lab or institution, cloud computing is an attractive option. Galaxy Cloud [217,218] allows users to run a private Galaxy installation on Amazon Web Services (AWS) elastic compute cloud (EC2) with the same functionalities as the main site using a virtual machine model. Omics pipe [219], an open source Python framework for automating multi-omics data analysis, is also available as Amazon virtual machine. XCMS online [220] is a cloud-based metabolomics data processing platform for predictive pathway analysis and enables multi-omics data analysis by integrating gene and protein data with metabolic pathways. MetaboAnalyst [221] is another cloud-based platform for integrative metabolomics analysis. It incorporates modules for multi-omics data integration through knowledge-based network analysis and various ML-based clustering, feature selection and classification algorithms. In addition to cloud-based bioinformatics platforms, machine learning-as-a-service is being offered by leading commercial cloud service providers like Amazon, Google, Microsoft and IBM. ML-as-a-service makes implementation of complex ML algorithms on large-scale datasets convenient for biomedical researchers [222]. It is apparent that the future of multi-omics integrative analysis is reliant on ML algorithms, and cloud-based solutions provide feasible options to implement them at large-scale.

## 7. Conclusions and Future Perspectives

High-throughput omics technologies are generating large volumes of multi-omics data at an unprecedented rate. Simultaneous analysis of data obtained from different platforms, for the same biological specimen, captures a holistic view of the complex biological interactions. For single-omics studies, traditional machine learning (ML) algorithms have been very successful in automatically identifying complex patterns from big data. However, multi-omics integrative analysis poses new computational challenges and amplifies the ones associated with single-omics studies. In this paper, we focused on five computational problems frequently encountered in integrative multi-omics data analysis, including the curse of dimensionality, data heterogeneity, missing data, rarity and class imbalance, and scalability issues. We reviewed some novel ML-based approaches recently applied to integrative analysis of multi-omics datasets, under each of the five problem categories. Furthermore, we also discussed state-of-the-art computational methods which have the potential to address these problems in multi-omics analysis. This article will help bioinformatics researchers in exploring modern computational approaches to tackle evolving challenges in integrative analysis. It also bridges the gap between problems in multi-omics integrative analysis, and novel machine learning approaches from the computer science community as potential solutions to these problems. Although this article addressed some key issues in integrative data analysis, there are other challenges that require attention in future studies. For example, specialized ML-based approaches need to be developed for multi-omics analysis in personalized medicine where cohort size can be very small (e.g., 100 patients or less) [223]. Moreover, additional machine learning frameworks which leverage prior knowledge of biological networks to integrate omics datasets should be proposed as they are vital for robust biomarker modelling [224,225,226]. In the integrative analysis of omics data and electronic health records (EHR) [227], or observational data and biomedical literature, sophisticated text mining and natural language processing approaches may play key roles to simultaneously handle structured and unstructured data [228,229,230]. However, the privacy and security of patient data should be ensured when developing ML approaches with EHRs and multi-omics. Integrative studies must comply with standards like Health Insurance Portability and Accountability Act (HIPPA) and any prediction or outcome from ML analysis must not compromise patient confidentiality. Collaborative studies can greatly benefit from privacy-preserving machine learning frameworks as institutions can jointly train accurate ML models without sharing sensitive patient data [231,232]. Finally, there is a need to benchmark ML methods for multi-omics analysis as numerous methods are available to solve the same problem. Although there are ongoing efforts to benchmark machine learning algorithms [233], benchmarking specific to multi-omics is required.
